# Post-Adoption Problem Behaviours in Adolescent and Adult Dogs Rehomed through a New Zealand Animal Shelter

**DOI:** 10.3390/ani8060093

**Published:** 2018-06-11

**Authors:** M. Carolyn Gates, Sarah Zito, Julia Thomas, Arnja Dale

**Affiliations:** 1 School of Veterinary Science, Massey University, Private Bag 11-222, Palmerston North 4442, New Zealand; c.gates@massey.ac.nz; 2 RNZSPCA, PO Box 15-309, New Lynn, Auckland 0640, New Zealand; arnja.dale@spca.nz; 3 Society for the Prevention of Cruelty to Animals Auckland, 50 Westney Rd, Mangere, Auckland 2022, New Zealand; foxx00@xtra.co.nz

**Keywords:** shelter medicine, adoption, dogs, behaviour, human-animal bond, animal welfare

## Abstract

**Simple Summary:**

Problem behaviours in dogs rehomed through animal shelters can jeopardise the long-term success of adoptions if not correctly managed. Data from 61 adolescent and adult dog adoptions that occurred through an animal shelter in Auckland, New Zealand, was analysed to identify the most common problem behaviours affecting adopted dogs and how concerned the new owners were about these problem behaviours. The majority of dogs had at least one reported problem behaviour; the most frequently reported problem behaviours were poor manners, destruction of household items, and excessively high energy. Very few dogs showed territorial aggression when objects or food items were removed, but aggression toward people or other dogs were both reported in nearly a fifth of dogs. The majority (87%) of adopters whose dog had some problem behaviours were not concerned at all or were a little concerned, and only three adopters were very concerned. Based on our interpretation of these findings, post-adoption support programmes targeted toward teaching adopters how to correctly train their dogs may be beneficial to increasing adoption satisfaction.

**Abstract:**

Problem behaviours in dogs rehomed through animal shelters can jeopardise the long-term success of adoptions. In this study, data from 61 adolescent and adult dog adoptions that occurred through an animal shelter in Auckland, New Zealand, from 1 November 2015 to 31 July 2016 were analysed to describe the frequency of problem behaviours and level of adopter concern at different time points post-adoption. Amongst the 57 dogs with behavioural information available, 40 (70%) had at least one reported problem behaviour, and the most frequently reported problem behaviours were poor manners (46%), destruction of household items (30%), and excessively high energy (28%). Very few dogs showed territorial aggression when objects or food items were removed (2% and 4%, respectively). However, aggression toward people or other dogs was frequently reported (19% and 19%, respectively). Of the 54 adopters that provided a response about their level of concern over their dog’s problem behaviours, 24 (44%) were not concerned at all, 23 (43%) were a little concerned, 4 (7%) were moderately concerned, and 3 (6%) were very concerned. Based on our interpretation of these findings, post-adoption support programmes targeted toward teaching adopters how to correctly train their dogs may be beneficial to increasing adoption satisfaction.

## 1. Introduction

The surrender of dogs to shelters is widespread, with many thousands of dogs surrendered every year [1,2]. While human-related factors such as moving to housing that does not allow dogs or changes in lifestyle that are incompatible with dog ownership are most often cited as reasons for surrender, behavioural issues can also play a significant role in preventing owners from bonding with their dogs [3,4,5,6,7]. This is a concern for animal shelters because previous research has suggested that dogs surrendered to a shelter for behavioural reasons are less likely to be rehomed [8], and those that are rehomed are more likely to be returned to the shelter as a failed adoption [9,10,11]. There are also significant concerns about danger to the community and the potential for liability issues with rehoming animals with known behavioural issues, particularly aggressive behaviour [12,13]. It has been suggested that potential adopters should be provided with support to better manage these problems and increase long-term adoption success [10,11]. 

Shelters use a variety of behavioural assessment tools to try and ensure that potentially dangerous dogs are not rehomed [14,15,16,17]. However, these have significant limitations, and it has been suggested that it is unlikely that problem behaviours after adoption can be reliably predicted using such assessments [15,18]. There have been several research studies looking at the prevalence of problem behaviours in dogs that were relinquished or returned to the shelter [3,5,9,10,11,19]. Common behavioural issues include fearfulness, excessive barking, hyperactivity, inappropriate toileting behavior, destructiveness, intolerance of other companion animals, straying, sexual behaviours, or poor manners [10,20]. However, the frequency of these behavioural issues varies significantly between studies and geographic locations, and there is little research that reports post-adoption behaviour problems in rehomed shelter dogs that remain with the adopter [18,20,21]. 

To the authors’ knowledge, behavioural issues displayed by dogs after adoption have not been previously reported in New Zealand. Knowledge of behavioural issues displayed by dogs after adoption can help to guide the provision of appropriate advice and support for adopters to try and improve human-dog bonding and adoption success. The objective of this study was to collect data on the prevalence of post-adoption behavioural issues in adolescent and adult dogs rehomed through an animal shelter in Auckland, New Zealand.

## 2. Materials and Methods 

The sampling frame for this study included all 108 adolescent and adult dog adoptions that occurred at an animal shelter in Auckland, New Zealand between 1 November 2015 and 31 July 2016. 

Information on the adopter contact details and the animal’s breed, sex, known or estimated age, entry date into the shelter, length of time spent in foster care (if any), and date of adoption were extracted from the electronic shelter records. This information was used to derive the animal’s approximate age at the time of adoption. For the purpose of this study, an adolescent or adult dog was defined as one with a known or estimated age of greater than six months. After reviewing the records, 10 dogs were excluded because they had been adopted by a shelter staff member, were part of a special investigation, or there was no valid e-mail address or phone number available for the adopter. This left a total of 98 adoptions for inclusion in the study.

A survey was developed based on previously published work [22,23]. In addition, relevant elements were incorporated from other authors’ questionnaires for evaluating behaviour problems, management factors and household composition [24,25,26,27,28], and the type and frequency of exercise [29]. The survey included questions on (1) how well the animal was adjusting to the new home, (2) how well the animal got along with other people, cats, and dogs in the household, (3) observed behavioural and anxiety problems, and (4) changes in behaviour since adoption. If the adopter no longer had the animal, he/she was asked questions about (1) the main reason for no longer having the animal and (2) the outcome for the animal. A copy of the complete survey is provided in the Supplementary Material.

The study aimed to collect data at one week, one month, three months, and six months post-adoption for each animal in the sampling frame. The survey was administered using e-mail invitations that asked adopters to complete the questions either through an online survey website or through a telephone interview with one of the researchers. The shelter had decided to implement a post adoption contact and support pilot programme and this was the primary reason for contacting the adopters. However, in order to assess and report on the pilot programme a standardised questionnaire was developed and data recorded on the responses. When the adopters were contacted they were told that the shelter was gathering information to investigate how well dogs were settling into their new homes and if there were any behavioural concerns, to determine whether post adoption support would be beneficial. At this time the adopters’ consent to participate in the study was requested and recorded. 

The staff member working on the project only worked part time; unfortunately, this resulted in inconsistencies in the number of invitations made and questionnaires completed at each time point because if the adopter could not be contacted on the staff member’s working days it was often another week until it was possible to try and contact the adopter again. As a result, some of the initial contacts were made two weeks post adoption and, consequently, it was then too soon to send another questionnaire two weeks later. In addition, the follow-up time was cut short due to a change in staffing reassigning the staff member undertaking the research and no one being available to take her place and finish the follow-ups.

Data on the length of time spent for each telephone interview were recorded to help estimate staff time requirements for providing post-adoption support to new dog adopters. Due to the significant time constraints encountered with conducting the survey by telephone, this option was largely discontinued four weeks into the study time period. However, adopters were still given the option of requesting additional follow-up by telephone using the online survey. It should also be noted that data collection continued only until 31 August 2016 meaning that adoptions occurring later in the sampling time frame were inherently right censored. The shelter records were reviewed six months after the study ended to identify any dogs that were returned to the shelter, which we classified as an adoption failure. 

Data collected via the telephone surveys were entered into the same online survey tool as the data collected via the adopters who completed the survey directly online and imported into the R statistical software package (version 3.4.1) for further processing and analysis [30]. Descriptive statistics were provided on the animal demographic characteristics, frequency of problem behaviours, and how well the owners believed the dog was settling in post-adoption. Data on the frequency of post-adoption problem behaviours was taken from the first completed survey for each dog, which included 10 surveys at one week post-adoption (16%), 22 surveys at one month post-adoption (36%), 19 surveys at three months post-adoption (31%) and 10 surveys at six months post-adoption (16%). Behavioural data from four respondents were incomplete, and these responses were discarded from subsequent analyses. A Fisher’s Exact test was used to determine if adopters with dogs that displayed aggression (biting at animals, biting at humans, and/or reacting aggressively when objects are removed) were more likely to be concerned than adopters with dogs that had no problem behaviours or non-aggressive problem behaviours. 

## 3. Results

### 3.1. Survey Response Rates

A total of 167 survey invitations were extended to the 98 eligible adopters at intervals between one week and six months post-adoption (Table 1). Data from at least one post-adoption interval was available for 61 dogs out of the 98 eligible adoptions (62%) with 25 of the 61 adopters (41%) completing surveys at two or more different time points. The total survey invitation response rate was, therefore, 53%. Out of the 89 completed surveys, data for 71 (80%) were submitted by the adopters online, and 18 (20%) were collected through telephone interviews. On average, each telephone interview took approximately 35 min to complete (range: 13 min to 58 min).

### 3.2. Animal Demographics

There were approximately 22 unique dog breeds represented in the study sample. The most common breed was Pitbull Terrier type dogs (18 out of 61, 30%), followed by Bull Mastiffs (5 out of 61, 8%). Small or toy breed dogs (including Miniature Pinschers, Jack Russell Terriers, Fox Terriers, Silky Terriers, Pomeranians, German Spitz, and Japanese Spitz) represented 15 out of 61 adopted dogs (25%). Female dogs (37 out of 61, 61%) outnumbered male dogs (24 out of 61, 39%), and the distribution by age category was 16 dogs from six to eleven months of age (26%), 22 dogs from 12 to 24 months of age (36%), 15 dogs from 25 to 71 months of age (25%), and 8 dogs from 72 months of age or older (13%). The average time from admission to the shelter until adoption was 117 days (median: 78 days, range: 18 days to 846 days). 

### 3.3. Post-Adoption Problem Behaviours

Overall, 47 out of 61 adopters (77%) reported that their dog was adjusting extremely well, 11 out of 61 adopters (18%) reported that their dog was adjusting moderately well, and 2 out of 61 adopted (3%) reported their dog was adjusting only fair or poorly. Only one adopter no longer had the dog (a female Labrador, approximately 1.5 years old) by three months post-adoption because the dog did not get along with other dogs in the household. 

Most adopters (33 out of 61, 54%) reported that their dog approached new people easily, but 15 out of 61 (25%) indicated that the dog was shy or fearful, and six out of 61 (10%) indicated that the dog did not like strangers. Although aggression toward household cats was reported in eight dogs (13%) and aggression toward unfamiliar dogs was reported in six dogs (10%), most dogs reportedly got on well with other animals (Table 2).

Amongst the 57 dogs with behavioural information available, 40 (70%) had at least one reported problem behaviour in the post-adoption period (Table 3). The most frequently reported problem behaviours were poor manners (e.g., jumping-up, pulling on leash) (46%), destruction of household items (30%), and excessively high energy (28%). Very few dogs showed possessive aggression when objects or food items were removed (2% and 4%, respectively). However, aggression towards people, or other dogs, was more frequently reported (19% and 19%, respectively). Out of the 54 adopters that provided a response about their level of concern about problem behaviours, 24 (44%) were not concerned at all, 23 (43%) were a little concerned, four (7%) were moderately concerned, and three (6%) were very concerned. Adopters with dogs that exhibited at least one aggressive behaviour had a significantly higher level of concern than other adopters based on a Fisher’s Exact test (*p* = 0.012). 

Overall, behaviours generally associated with separation anxiety were reported to occur never or rarely in the 54 dogs with complete data (Table 4). When asked about the level of concern about separation-related problems, 39 (72%) adopters were not concerned at all, eight (15%) were a little concerned, four (7%) were moderately concerned, and three (6%) were very concerned.

Longitudinal data were available for 25 dogs with complete surveys for at least two post-adoption time periods. For 13 out of 25 dogs (52%), there was no change in the number of reported problem behaviours between surveys (Table 5). Problem behaviours resolved completely for five out of the 25 dogs (20%), decreased in frequency for two out of the 25 dogs (8%), and increased in frequency for five out of the 25 dogs (20%).

## 4. Discussion

More than two-thirds of rehomed animals included in this study had at least one reported problem behaviour in the post-adoption period. This is consistent with the prevalence reported in other comparable studies; Wells and Hepper [20] found that 68.3% of respondents reported that their dog adopted from a shelter exhibited at least one behaviour problem within the first month, and Lord et al. [21] reported 67.9% of respondents. Similar to other studies, poor manners, destructive behaviour, and excessively high energy were commonly reported problems in the current study [20,21,24,31]. The prevalence of toileting problems in our study was lower than that in previous studies of rehomed dogs. This could be explained by the fact that the other studies included puppies [20,21], whereas in the current study, only dogs over six months of age were included and, presumably, adult dogs are less likely to display toileting problems compared to puppies. Separation-related problems were also less commonly reported in our current study compared with previous studies of dogs obtained from shelters [24,25,32]; in other similar studies, separation-related problems were reported in 34% [24], 16.8% [25], and 30% [32] of dogs. However, even though most other post-adoption assessments of the prevalence of behaviour problems were also made via owner questionnaires, direct comparison is difficult as separation-related problems were reported in different ways in different studies (for example, listing different separation-related behaviours or having a single reporting category for separation anxiety). However, other researchers have shown that video footage of dog’s behaviour when left alone correlates well with owner reports of separation-related problems [33,34] and also that owner reports of fear related behaviour corresponded well to physical behavioural tests in dogs [29], indicating that owner reporting of behaviours are potentially a relatively accurate measurement. Additionally, in the current study, the dogs’ problem behaviours were owner-reported through simple survey questions, which do not provide sufficiently detailed and verified information to make an accurate behavioural diagnosis.

Aggression-related behaviours toward dogs or people were reported for 19% of dogs in our study, which falls within ranges reported by other published studies. For example, Mornement et al. [18] reported that 10.8% of adopted dogs showed aggression toward animals often or very often, and 24.3% of the adopted dogs in that study had growled, snapped at, or attempted to bite a person. Wells and Hepper reported that 8.9% of adopted dogs showed aggression toward dogs and 5.5% toward humans [20], Lord et al. [21] reported that 14.9% of dogs showed biting, growling, or snapping at people or animals, and Scott et al. [35] reported that 21.1% of adopted dogs showed aggression toward dogs and 5.3% toward humans. The relatively high prevalence of aggression toward dogs and people is of concern, since aggression may pose a risk to the community and has also been associated with an increased risk of an adoption being unsuccessful [9]. In the current study, adopters of dogs with aggressive behaviours had significantly higher levels of concern about problem behaviours than adopters of dogs with non-aggressive problem behaviours, which suggests the need for animal shelters to follow-up when possible with adopters to provide support for managing behaviours. Unfortunately, there was no available information on problem behaviours of dogs at the time of admission or during their stay in the shelter and too small a sample size to make inferences about other risk factors for aggression. In future studies, it would be useful to determine if the problem behaviours after adoption are the same as those reported by people who surrender the animal or whether time spent in the shelter increases the risk of animals developing certain problem behaviours.

Poor manners (generally termed control problems in the literature) and other behaviour problems may contribute to reduced attachment between the adopter and the dog, poor satisfaction with the adopter’s relationship with the dog, and might ultimately lead to relinquishment [36,37,38,39]. Poor manners and other behaviour issues were commonly reported in adopted dogs in this study, highlighting the importance of ensuring adopters have realistic expectations, and have the support they need to address any problems. There is scope for more/better training in shelter to improve dogs’ ‘manners’ prior to adoption, which is likely to have benefits beyond those experienced after adoption as it has been shown that training of shelter dogs increases their chances of being adopted [40]. However, support for new owners is paramount [11], particularly if adult dogs have practiced particular undesirable behaviours for long periods of time prior to their relinquishment to the shelter, or there have been deficits during sensitive periods of their development [41], as it is unlikely a relatively short period of training in a shelter environment will resolve the issues. Providing new adopters with more information about ongoing training to foster better relationships, and opportunities to access behavioural support, is likely to increase owner satisfaction with their new animal [11].

Although a relatively high percentage of the dogs in our study did have some problem behaviours, there was only one reported adoption failure, which occurred because the rehomed dog did not get on with the other household dogs. We cannot rule out the possibility that the adoption failure rate was higher in non-responders or that more dogs were returned to the shelter after the study finished. However, given that most adoption failures tend to occur within one month, and often within two weeks, of the adoption, this is unlikely to have significantly biased the results [9,11,36,42]. For most shelter organisations, it is difficult to accurately assess adoption failure since adopters may utilise other avenues for rehoming dogs such as giving the dog to a friend or family member, rehoming the dog through another rescue or sheltering organisation, or attempting to rehome the dog privately [36,43], rather than returning the dog to the original shelter. Further, our study excluded rehomed puppies under the age of six months, which might further have underestimated our reported return rate.

In the present study, many of the problem behaviours reported in dogs with multiple follow-up points had not resolved, which may indicate the need for more long-term support to adopters. Various pre- and post- adoption interventions have been assessed to see if they confer any advantage in terms of reducing undesirable behaviours and adoption failures, although research in this area has been limited to date and there is no conclusive evidence as to which interventions are most effective [2,36]. For example, encouraging walking of newly adopted dogs by their new owners was not found to be associated with the success of the adoption [36] and the provision of pre-adoption counseling, written information, and a food toy was not found to reduce the incidence of separation anxiety in adopted dogs [25]. However, providing pre-adoption counseling on housetraining was reported to increase housetraining success [25] and providing written advice aimed at reducing the occurrence of separation-related behaviour problems after rehoming was reportedly associated with fewer problems [32]. Some research has suggested that attending training (particularly for puppies) and behavioural counseling results in adopters feeling a stronger bond with their dog and the dogs having better manners and fewer behavioural issues [44,45] and may be associated with a lower risk of surrender of the adopted dog [6]. The surrender of dogs showing aggressive behaviours is reported to be less likely if their owners sought advice from the animal shelter [9]. Foster care of dogs prior to adoption where the foster carers were also involved with rehoming the dogs has been associated with lower return rates compared to dogs rehomed directly from the shelter [46]. Our sample did include adolescent dogs as well as adult dogs and it must be acknowledged that pre-pubertal adolescent dogs of 6–10 months of age could behave differently than older post-pubertal dogs. Unfortunately, although it would have been ideal to understand more about the risk factors for the different separation anxiety–type behaviours and whether these were different for adolescent versus adult dogs, there were not enough cases of each when the data were stratified to permit robust statistical analysis. Therefore, the decision was made to exclude these analyses rather than risk over-interpretation of the findings of such an analysis. In future studies, a larger number of adolescent and adult dogs could allow improved analysis and understanding of the risk factors for the different separation anxiety–type behaviours in adolescent and adult dogs.

Overall, adopter participation in the current study (62%) was similar to that reported by Blackwell et al. [32] (68%) but less than that reported by Elliott et al. [22] (79%) and Herron et al. [25] (87%). Considering the questionnaire response rates at each time point, the response rate in the current study (64%) at 1-month post-adoption was lower than reported by Herron et al. [25] and Elliott et al. [22], but higher than that reported by Wells and Hepper [20] (37%). At the 3-month time point, the response rate in the current study (58%) was substantially lower than reported by Blackwell et al. [32], and was lower again at the 6-month time point (41%). The relatively high participation in the study by Herron et al. [25] likely reflects the method of engagement, which was via telephone interview, and also possibly the relatively short questionnaire, which contained 14 questions. This is compared to the 31 questions in the current study’s questionnaire. Blackwell et al. [32] and Elliott et al. [22] sent their questionnaires (of 47 and 45 questions respectively) via normal post, whereas the questionnaires in the current study were predominantly sent via email. Whether the difference in delivery method had an effect on the response rate is unknown. An improvement for future research with surveys administered at multiple time points might be to have a shorter version of the questionnaire for subsequent time points, or a more concise questionnaire. It would also be useful to validate how well the simple survey questions reflect the dog’s true behaviour in the new home to know whether it is a reliable method for assessing behaviour.

Another limitation of the data collected relates to the inconsistency of when the behavioural data was collected (i.e., one week to six months). Post adoption behaviour is likely to change over this time period as the dog settles in to his or her new home and routine [22]. Furthermore, just because an owner may not be concerned about problem behaviours in their dog does not mean that problem behaviours are not actually present. In future research, more consistent data collection time intervals should be attempted, and this factor could also be included in statistical models to formally test risk factors for post-adoption outcomes. In addition, the high variation in the breeds of dogs included in the study (22 different types) probably had some effect on the results in terms of post adoption behaviour since different breeds have individual behaviour traits [47,48]. This large variation in breed is likely unavoidable in shelter based research (and was seen in another similar study where breed was reported [35]), but a larger sample size would allow analysis of the potential effects of breed on behaviour.

The average time taken to conduct the telephone interview (35 min) was considerably longer than the estimated time required to complete the online questionnaire (15 min). The additional time taken for telephone interviews was due to the dual nature of the project, which was to provide post-adoption support, as well as to gather post-adoption data. The shelter does not currently have dedicated resources, or approved external providers, for professional animal behaviour support for adopters (shelter staff are permitted to refer adopters to their local veterinarian for recommendations on training and behaviour providers in their area). Therefore, where adopters wished to discuss aspects of their dog’s behaviour, time was taken to investigate the behaviour further and to provide telephone support or additional resources as required. Also, it was not uncommon for adopters to elaborate beyond the scope of the actual question. Other researchers conducting post-adoption surveys (for example, Blackwell et al. [32] and Herron et al. [25]) have been able to refer adopters who identified behaviours of concern to professional animal behaviour support within the adopting organisation, thereby keeping the data collection and post-adoption support separate. This approach would be preferable.

It seems likely, based on the current evidence, that a combination of pre-adoption measures (such as utilising foster care when possible, pre-adoption counseling, and providing written information on certain behaviour issues) and post-adoption measures (such as offering training, particularly for puppies, and support/behavioural counseling for adopters of dogs with problem behaviours) will result in the best outcomes for adopters and their dogs. However, more research is needed to provide conclusive evidence in this area. Provision of post-adoption follow-up and support in this study required substantial investment of resources. Therefore, it may also be worth assessing greater use of technologies to help provide post-adoption support in a less resource intensive manner; this could include the use of short-message service (SMS), emails, social media platforms and groups, and other emerging technologies. This could both reduce the cost of post-adoption support services provision and also engage with a wider range of adopters in a convenient and helpful way.

## 5. Conclusions

In the current study, many adopted dogs had least one reported problem behaviour, the most frequently reported were poor manners, destruction of household items, and excessively high energy. Almost one fifth of dogs showed aggression toward people or other dogs. Most adopters were not concerned over their dog’s problem behaviours, but adopters of dogs with aggressive behaviours had higher levels of concern about the behaviours than adopters of dogs with non-aggressive problem behaviours. It seems prudent for animal shelters to follow-up when possible with adopters to provide support for managing problem behaviours and post-adoption support programmes targeted toward teaching adopters how to correctly train their dogs may be beneficial to increasing adoption satisfaction. 

## Figures and Tables

**Table 1:** Response rates of individuals, who adopted adult dogs from the participating shelter, to surveys administered at one week, one month, three months, and six months post-adoption.

Response Rate Categories	One Week	One Month	Three Months	Six Months	Total
Total number of invitations to participate	24	42	62	39	167
Number of surveys completed by telephone	5	7	4	2	18
Number of surveys completed by e-mail link	5	20	32	14	71
Total number of surveys completed	10	27	36	16	89
Final response rate (%)	42%	64%	58%	41%	53%

**Table 2:** Responses of 61 adopters to survey questions about how well adult dogs rehomed by the participating animal shelter in Auckland, New Zealand, get along with other animals.

Survey Questions about How Well Dogs Get Along with Other Animals	Unfamiliar Dogs N (%)	Familiar Dogs N (%)	Cats N (%)
Hasn’t met yet/Do not own	6 (10%)	39 (64%)	28 (46%)
Gets along well	37 (61%)	17 (28%)	9 (15%)
Uninterested	4 (7%)	2 (3%)	3 (5%)
Attacks or chases	6 (10%)	1 (2%)	8 (13%)
Afraid of	5 (8%)	1 (2%)	7 (11%)
Other response	3 (5%)	1 (2%)	6 (10%)

**Table 3:** Number and type of problem behaviours reported in a survey administered to 57 adopters of adult dogs rehomed by the participating animal shelter in Auckland, New Zealand. ^1^

Problem Behaviour	Number	%
Total number of problem behaviours (out of 13 assessed) ^2^		
None	17	30%
1	16	28%
2	11	19%
3	4	7%
4 or more	9	16%
Has your dog shown any of the following behavioural problems? ^2^		
Aggression towards other animals	11	19%
Aggression towards people	11	19%
Destructive towards household items	17	30%
Dislikes being physically handled	2	4%
Excessive or high energy	16	28%
House training or toileting problems	9	16%
Poor manners	26	46%
Noisy or barking while someone is home	4	7%
Escaping the property	3	5%
Runs away or does not comes when called off leash	10	18%
Does not respond to training corrections	4	7%
Aggressive over having objects removed	1	2%
Aggressive over have food items removed	2	4%

^1^ Based on the first survey completed by adopters. ^2^ Complete data were available for 57 out the 61 adopters.

**Table 4:** Responses of adopters to survey questions about the frequency of separation-related behaviours in 54 adult dogs rehomed through the participating animal shelter in Auckland, New Zealand.

Survey Questions about Separation-Related Behaviours	Never	Rarely	Sometimes	Often	Always	Don’t Know
	n (%)	n (%)	n (%)	n (%)	n (%)	n (%)
Has your dog shown any of the following separation-related behaviours?						
Barking or whining for long periods ^a^	26 (48%)	12 (22%)	5 (9%)	1 (2%)	1 (2%)	9 (17%)
Destruction of property ^a^ (chewing, digging, or scratching)	26 (48%)	11 (20%)	11 (20%)	1 (2%)	2 (4%)	3 (6%)
Self-injurious behavior ^a^ (e.g., over-grooming)	43 (80%)	7 (13%)	0 (0%)	0 (0%)	1 (2%)	3 (6%)
Escaping from the property ^a^	39 (72%)	10 (19%)	2 (4%)	0 (0%)	0 (0%)	3 (6%)
Overly excited when owner leaves the property	29 (54%)	14 (26%)	8 (15%)	1 (2%)	0 (0%)	2 (4%)
Overly excited when owner returns to the property	16 (30%)	11 (20%)	12 (22%)	8 (15%)	5 (9%)	2 (4%)

^a^ Only in the absence of the owner (i.e., when the dog is left alone).

**Table 5:** Change in the number of problem behaviours reported by adopters of 25 adult dogs, rehomed through the participating animal shelter in Auckland, New Zealand between two survey time points.

		Survey 2						
		0	1	2	3	4	5	6
**Survey 1**	0	5	1					1
	1	1	3	2				
	2	1		2			1	
	3	1	1		2			
	4					1		
	5	1						
	6	1	1					

## Supplementary Materials

The following are available online at http://www.mdpi.com/2076-2615/8/6/93/s1: Post-adoption survey for adult dogs.

## References

[1] Weiss E., Gramann S., Victor Spain C., Slater M. (2015). Goodbye to a Good Friend: An Exploration of the Re-Homing of Cats and Dogs in the U.S. Open J. Anim. Sci..

[2] Coe J.B., Young I., Lambert K., Dysart L., Nogueira Borden L., Rajić A. (2014). A Scoping Review of Published Research on the Relinquishment of Companion Animals. J. Appl. Anim. Welf. Sci..

[3] Salman M.D., New J., Scarlett J.M., Kass P.H., Ruch-Gallie R., Hetts S. (1998). Human and Animal Factors Related to Relinquishment of Dogs and Cats in 12 Selected Animal Shelters in the United States. J. Appl. Anim. Welf. Sci..

[4] New J.C., Kelch W.J., Hutchison J.M., Salman M.D., King M., Scarlett J.M., Kass P.H. (2004). Birth and death rate estimates of cats and dogs in U.S. households and related factors. J. Appl. Anim. Welf. Sci..

[5] Salman M.D., Hutchison J., Ruch-Gallie R., Kogan L., New J.C., Kass P.H., Scarlett J.M. (2000). Behavioral Reasons for Relinquishment of Dogs and Cats to 12 Shelters. J. Appl. Anim. Welf. Sci..

[6] Patronek G.J., Glickman L.T., Beck A.M., McCabe G.P., Ecker C. (1996). Risk factors for relinquishment of dogs to an animal shelter. J. Am. Vet. Med. Assoc..

[7] Weiss E., Gramann S., Drain N., Dolan E., Slater M. (2015). Modification of the Feline-Ality^TM^ Assessment and the Ability to Predict Adopted Cats’ Behaviors in Their New Homes. Animals.

[8] Lepper M., Kass P.H., Hart L.A. (2002). Prediction of adoption versus euthanasia among dogs and cats in a California animal shelter. J. Appl. Anim. Welf. Sci..

[9] Diesel G., Pfeiffer D.U., Brodbelt D. (2008). Factors affecting the success of rehoming dogs in the UK during 2005. Prev. Vet. Med..

[10] Mondelli F., Prato Previde E., Verga M., Levi D., Magistrelli S., Valsecchi P. (2004). The bond that never developed: Adoption and relinquishment of dogs in a rescue shelter. J. Appl. Anim. Welf. Sci..

[11] Shore E.R. (2005). Returning a Recently Adopted Companion Animal: Adopters’ Reasons for and Reactions to the Failed Adoption Experience. J. Appl. Anim. Welf. Sci..

[12] Marston L., Bennett P., Rolf V., Mornement K. (2008). Review of Strategies for Effectively Managing Unwanted Dogs and Cats in Queensland. A Report to the Department of Primary Industries and Fisheries, Queensland.

[13] Barnard S., Siracusa C., Reisner I., Valsecchi P., Serpell J.A. (2012). Validity of model devices used to assess canine temperament in behavioral tests. Appl. Anim. Behav. Sci..

[14] Mornement K.M., Coleman G.J., Toukhsati S., Bennett P.C. (2010). A review of behavioral assessment protocols used by australian animal shelters to determine the adoption suitability of dogs. J. Appl. Anim. Welf. Sci..

[15] Patronek G.J., Bradley J. (2016). No better than flipping a coin: Reconsidering canine behavior evaluations in animal shelters. J. Vet. Behav..

[16] Bollen K.S., Horowitz J. (2008). Behavioral evaluation and demographic information in the assessment of aggressiveness in shelter dogs. Appl. Anim. Behav. Sci..

[17] Bennett S.L., Litster A., Weng H.Y., Walker S.L., Luescher A.U. (2012). Investigating behavior assessment instruments to predict aggression in dogs. Appl. Anim. Behav. Sci..

[18] Mornement K.M., Coleman G.J., Toukhsati S.R., Bennett P.C. (2015). Evaluation of the predictive validity of the Behavioural Assessment for Re-homing K9’s (B.A.R.K.) protocol and owner satisfaction with adopted dogs. Appl. Anim. Behav. Sci..

[19] New J.C., Salman M.D., King M., Scarlett J.M., Kass P.H., Hutchison J.M. (2000). Characteristics of Shelter-Relinquished Animals and Their Owners Compared With Animals and Their Owners in U.S. Pet-Owning Households. J. Appl. Anim. Behav. Sci..

[20] Wells D.L., Hepper P.G. (2000). Prevalence of behaviour problems reported by owners of dogs purchased from an animal rescue shelter. Appl. Anim. Behav. Sci..

[21] Lord L.K., Reider L., Herron M.E., Graszak K. (2008). Health and behavior problems in dogs and cats one week and one month after adoption from animal shelters. J. Am. Vet. Med. Assoc..

[22] Elliott R., Toribio J.-A.L.M.L., Wigney D. (2010). The Greyhound Adoption Program (GAP) in Australia and New Zealand: A survey of owners’ experiences with their greyhounds one month after adoption. Appl. Anim. Behav. Sci..

[23] Miller L., Zawistowski S. (2013). Shelter Medicine for Veterinarians and Staff.

[24] Blackwell E.J., Twells C., Seawright A., Casey R.A. (2008). The relationship between training methods and the occurrence of behavior problems, as reported by owners, in a population of domestic dogs. J. Vet. Behav. Clin. Appl. Res..

[25] Herron M.E., Lord L.K., Husseini S.E. (2014). Effects of preadoption counseling on the prevention of separation anxiety in newly adopted shelter dogs. J. Vet. Behav. Clin. Appl. Res..

[26] McGreevy P.D., Masters A.M. (2008). Risk factors for separation-related distress and feed-related aggression in dogs: Additional findings from a survey of Australian dog owners. Appl. Anim. Behav. Sci..

[27] Storengen L.M., Boge S.C.K., Strøm S.J., Løberg G., Lingaas F. (2014). A descriptive study of 215 dogs diagnosed with separation anxiety. Appl. Anim. Behav. Sci..

[28] Takeuchi Y., Ogata N., Houpt K.A., Scarlett J.M. (2001). Differences in background and outcome of three behavior problems of dogs. Appl. Anim. Behav. Sci..

[29] Tiira K., Lohi H. (2015). Early life experiences and exercise associate with canine anxieties. PLoS ONE.

[30] R-Development-Core-Team (2016). R: A Language and Environment for Statistical Computing.

[31] Hennessy M.B., Voith V.L., Mazzei S.J., Buttram J., Miller D.D., Linden F. (2001). Behavior and cortisol levels of dogs in a public animal shelter, and an exploration of the ability of these measures to predict problem behavior after adoption. Appl. Anim. Behav. Sci..

[32] Blackwell E.J., Casey R.A., Bradshaw J.W.S. (2016). Efficacy of written behavioral advice for separation-related behavior problems in dogs newly adopted from a rehoming center. J. Vet. Behav. Clin. Appl. Res..

[33] Konok V., Dóka A., Miklósi Á. (2011). The behavior of the domestic dog (Canis familiaris) during separation from and reunion with the owner: A questionnaire and an experimental study. Appl. Anim. Behav. Sci..

[34] Van Rooy D., Arnott E.R., Thomson P.C., McGreevy P.D., Wade C.M. (2018). Using an owner-based questionnaire to phenotype dogs with separation-related distress: Do owners know what their dogs do when they are absent?. J. Vet. Behav. Clin. Appl. Res..

[35] Scott S., Jong E., McArthur M., Hazel S.J. (2018). Follow-up surveys of people who have adopted dogs and cats from an Australian shelter. Appl. Anim. Behav. Sci..

[36] Gunter L., Protopopova A., Hooker S.P., Der Ananian C., Wynne C.D.L. (2017). Impacts of Encouraging Dog Walking on Returns of Newly Adopted Dogs to a Shelter. J. Appl. Anim. Welf. Sci..

[37] Marston L.C., Bennett P.C., Coleman G.J. (2004). What happens to shelter dogs? An analysis of data for 1 year from three Australian shelters. J. Appl. Anim. Welf. Sci..

[38] Kwan J.Y., Bain M.J. (2013). Owner Attachment and Problem Behaviors Related to Relinquishment and Training Techniques of Dogs. J. Appl. Anim. Welf. Sci..

[39] Serpell J.A. (1996). Evidence for an association between pet behavior and owner attachment levels. Appl. Anim. Behav. Sci..

[40] Luescher A.U., Tyson Medlock R. (2009). The effects of training and environmental alterations on adoption success of shelter dogs. Appl. Anim. Behav. Sci..

[41] McMillan F.D., Duffy D.L., Serpell J.A. (2011). Mental health of dogs formerly used as “breeding stock” in commercial breeding establishments. Appl. Anim. Behav. Sci..

[42] Protopopova A., Gunter L.M. (2017). Adoption and relinquishment interventions at the animal shelter: A review. Anim. Welf..

[43] Weiss E., Slater M., Garrison L., Drain N., Dolan E., Scarlett J.M., Zawistowsk S.L. (2014). Large dog relinquishment to two municipal facilities in New York city and Washington, D.C.: Identifying targets for intervention. Animals.

[44] Clark G.I., Boyer W.N. (1993). The effects of dog obedience training and behavioural counselling upon the human-canine relationship. Appl. Anim. Behav. Sci..

[45] Duxbury M.M., Jackson J., Line S.W., Anderson R.K. (2003). Evaluation of association between retention in the home and attendance at puppy socialization classes. J. Am. Vet. Med. Assoc..

[46] Mohan-Gibbons H., Weiss E., Garrison L., Allison M. (2014). Evaluation of a novel dog adoption program in two US communities. PLoS ONE.

[47] Sundman A.S., Johnsson M., Wright D., Jensen P. (2016). Similar recent selection criteria associated with different behavioural effects in two dog breeds. Genes Brain Behav..

[48] Svartberg K., Svartberg K. (2002). Personality traits in the domestic dog (Canis familiaris). Appl. Anim. Behav. Sci..

