# Supplementary material for: Post-Adoption Problem Behaviours in Adolescent and Adult Dogs Rehomed through a New Zealand Animal Shelter
Source: Animals (Basel). 2018 Jun 11;8(6):93. doi: 10.3390/ani8060093 (PMC6024916; doi:10.3390/ani8060093)
Supplement: Supplementary file 1 [file animals-08-00093-s001.pdf]

1. Date of adoption

Date

DD/MM/YYYY

2. Adopter's details

Name

3. Dog's details

Name

Breed

Gender

Age (estimate)

* 4. Generally, how well do you feel your new dog is adjusting to your home?

- ○ Extremely well
- ○ Moderately well
- ○ Fair
- ○ Poorly
- ○ Not working out well
- ○ I no longer have the dog

5. What is the main reason you no longer have the dog?

- ◯ Allergic to the dog
- ◯ Dog ran away or was stolen
- ◯ Behaviour problems
- ◯ Didn't get along with other pet(s)
- ◯ Health problems
- ◯ Moving and unable to take with me
- ◯ Not a good match for my family / lifestyle
- ◯ Unable to keep or care for / change in circumstances

Comments

6. What happened to the dog or where is it now?

- ◯ Returned to the SPCA
- ◯ Relinquished to another animal shelter or rehoming center
- ◯ Rehomed with a friend or relative
- ◯ Rehomed with an unrelated person
- ◯ Unknown e.g. lost, escaped or stolen
- ◯ Died or euthanised
- ◯ Other (please specify)

7. When did the dog leave your home?

Date

DD/MM/YYYY

Although you no longer have this dog, we would appreciate it if you would still complete this survey based on your experience during the time he/she was present in your home.

* 8. Do you have another dog(s) in the household? If so, how does your new dog generally get along with your other dog(s)?

- ◯ I don't have another dog
- ◯ Gets on well (e.g. approaches / plays well) with other dog
- ◯ Not that interested in other dog
- ◯ Afraid of other dog
- ◯ Barks, lunges or fights with other dog
- ◯ Hasn't met other dog yet
- ◯ Other (please specify below)

Please state whether you are concerned about your dog's behaviour in relation to your other dog(s) (yes, concerned / no, not concerned), and any additional comments

* 9. How does your dog generally get along with other dogs (not in the household)?

- ◯ Gets on well (e.g. approaches / plays well) with other dogs
- ◯ Not that interested in other dogs
- ◯ Afraid of other dogs
- ◯ Barks, lunges or fights with other dogs
- ◯ Hasn't met other dogs yet
- ◯ Other (please specify below)

Please state whether you are concerned about your dog's behaviour in relation to other dogs (yes, concerned / no, not concerned), and any additional comments

* 10. How does your dog seem to feel about other people he/she meets?

- Loves everyone, approaches happily
- Shy or fearful with other people
- Doesn't like strangers
- Doesn't like children
- Doesn't like some household adults
- Has not met any other people yet
- Other (please specify below)

Please state whether you are concerned about your dog's behaviour in relation to meeting other people (yes, concerned / no, not concerned), and any additional comments

11. How does your dog play with people?

- Very nice to play with
- Mouthy when playing
- Gets too rough and won't calm down
- Doesn't really want to play
- Other (please specify below)

Please state whether you are concerned about your dog's behaviour in relation to playing with people (yes, concerned / no, not concerned), and any additional comments

* 12. Do you have a cat? If so, how does your dog get along with your cat?

- I don't have a cat
- Loves the cat
- Not that interested in the cat
- Afraid of the cat
- Wants to chase / attack the cat
- Has not met the cat yet
- Other (please specify below)

Please state whether you are concerned about your dog's behaviour in relation to your cat (yes, concerned / no, not concerned), and any additional comments

* 13. Do you have 'other' pets (e.g. Rabbit, Guinea pig, Birds)? If so, how does your dog get along with your other pet(s)?

- I don't have 'other' pets
- Loves other pet(s)
- Not that interested in other pet(s)
- Afraid of other pet(s)
- Wants to chase / attack other pet(s)
- Has not met other pet(s) yet
- Other (please specify below)

Please state what type of ther pet(s) you have and whether you are concerned about your dog's behaviour in relation to your 'other' pet(s) (yes, concerned / no, not concerned).

14. How many dog(s) have you owned before?

As a child

As an adult

15. What type (breed) of dog(s) have you owned before?

As a child

As an adult

16. Have you, or are you planning to introduce training activities with your dog?

- Yes, just training at home
- Yes, joined (or signed up for) a class
- Yes, seeing (or signed up to see) a trainer
- No, but planning to
- No, not planning to

Comments (optional)

17. If you are doing some training with your dog, how do you feel he/she is getting on with the training?

- I haven't done any training yet
- Excellent, doing really well
- Good, doing well
- Fair, doing okay
- Poor, not doing that well
- Terrible

Comments (optional)

18. What kind of exercise does your dog get generally? And how often? (please choose a response from all three columns)

	How long do you spend on this activity at a time, on average?	How many times per day?	How many days per week or times per month?
Playing or training with you on your property			
Walking on a leash (off your property)			
Off leash exercise (off your property)			
Other activities off your property e.g. obedience, agility, tracking etc.			

Other (or comments) e.g. dog exercises itself on the property

* 19. How attached do you feel to your adopted dog?

- Strongly attached
- Moderately attached
- Mildly attached
- Not attached at all

Comments (optional)

20. How much time does your dog generally spend alone each day / each week?

	Number of days per week (should add up to seven)
Never left alone	
Less than one hour alone	
1 to 4 hours alone	
4 to 7 hours alone	
7 to 10 hours alone	
More than 10 hours alone	

Comments (optional)

* 21. During the day, when you are NOT home, is your dog generally (mainly)

- Indoors loose
- Indoors in a crate
- Indoors contained e.g. playpen, one room
- Indoors in a garage
- Outdoors loose on the property
- Outdoors tied up
- Outdoors contained e.g. a kennel and run
- Able to move between indoors and outdoors
- Other (please specify)

* 22. During the day, when you ARE AT HOME, is your dog generally (mainly)?

- Indoors loose
- Indoors in a crate
- Indoors contained e.g. playpen, one room
- Indoors in a garage
- Outdoors loose on the property
- Outdoors tied up
- Outdoors contained e.g. in a kennel and run
- Able to move between indoors and outdoors
- Other, or a combination (please specify)

23. Where does your dog sleep during the night?

- ◯ In a bedroom
- ◯ Elsewhere in the house
- ◯ In the garage
- ◯ Outside
- ◯ Other (please specify)

24. What does your dog sleep on or in at night?

- ◯ On your (or another family member's) bed
- ◯ On a dog bed
- ◯ On a sofa
- ◯ In a crate (inside)
- ◯ In a kennel (outside)
- ◯ Other (please specify)

* 25. Has your dog shown any of the following behaviour problems since he/she joined your family? Please tick all that apply.
Note: there is another question that deals with behaviour problems when your dog is left alone

- [ ] Biting, growling or snapping AT OTHER ANIMALS
- [ ] Biting, growling or snapping AT PEOPLE
- [ ] Chewing, digging or damaging objects he shouldn't (while someone is home)
- [ ] Dislikes being held, groomed or physically handled
- [ ] Excessive or high energy level
- [ ] House-training or toileting problems (e.g. eats own poo) while someone is home
- [ ] Poor manners (e.g. stealing food, jumping up, attention seeking, pulling on leash etc.)
- [ ] Noisy (when someone is home)
- [ ] Escaping the property (running away or fence jumping) while someone is home
- [ ] Running away / not coming when called when off leash
- [ ] Does not respond to corrections for unacceptable behaviour and repeats the behaviour
- [ ] Responds negatively (e.g. growls, lunges or snaps) when having toys / objects taken away from him/her
- [ ] Shows guarding or aggressive behaviour (e.g. growls, lunges or snaps) around food items, or if you try to touch or remove food from his/her bowl
- [ ] Has not shown any behaviour problems
- [ ] Other (please specify) or comments

* 26. If you identified problem behaviours in the previous question, how concerned are you about these behaviours?

- ( ) No behaviours of concern
- ( ) A little concerned
- ( ) Moderately concerned
- ( ) Very concerned

Comments (optional)

* 27. Some dogs show signs of anxiety when left alone, even for short periods of time. How often has your dog shown each of the following behaviours **when he / she is left alone** (or separated from you or other family members) since joining your family?

	Always or almost always	Most of the time	Some of the time	Rarely	Never	Not applicable / Don't know
Barking or whining for long periods when you are not home (this might be based on comments from your neighbours)	○	○	○	○	○	○
Destructiveness - chewing, digging or scratching at doors, gates, floor, windows, curtains etc.	○	○	○	○	○	○
Toileting inside (urination and/or defecation)	○	○	○	○	○	○
Self-injurious behaviour e.g. over-grooming (licking or nibbling him/herself excessively)	○	○	○	○	○	○
Escaping the property (e.g. fence jumping / running away)	○	○	○	○	○	○
Overly excited or concerned when you are about to leave e.g. panting, pacing, lip-licking, whining, constant following	○	○	○	○	○	○
Overly excited or concerned when you return e.g. panting, pacing, lip-licking, whining, constant following	○	○	○	○	○	○

Other (please specify) or comments

* 28. If you identified any separation related behaviours in the previous question, how concerned are you about those behaviours?

- ◯ No behaviours of concern
- ◯ A little concerned
- ◯ Moderately concerned
- ◯ Very concerned

Comments (optional)

29. Many dogs become strongly attached to their people, and some demand a great deal of attention and affection from them. How often has your dog shown each of the following behaviours?

	Always / almost always	Most of the time	Some of the time	Rarely	Never	Not applicable / Don't know
Tends to follow you (or other members of the family) from room to room	◯	◯	◯	◯	◯	◯
Tends to sit close to, or in contact with, you (others in the family) when you are sitting down	◯	◯	◯	◯	◯	◯

* 30. How has your dog's behaviour changed since coming to your home?

- ◯ Greatly improved
- ◯ Somewhat improved
- ◯ Staying the same
- ◯ Somewhat worse
- ◯ Much worse

* 31. Have you had to take your dog to the vet since the adoption?

- ◯ Yes
- ◯ No

* 32. What have you taken your dog to the vet for?

- ◯ Routine health check or vaccinations
- ◯ Minor injury
- ◯ Minor illness
- ◯ Other (please specify)

* 33. What do you feed your dog?

* 34. How often do you feed your dog?

- Once a day
- Twice a day
- Three times a day
- On demand
- Other (please specify)

35. Is there anything else you would like to comment on?

* 36. Are you happy for us to follow-up again with you?

- yes
- no thanks

Thank you very much for taking the time to complete this survey.
If you have any questions or concerns regarding your dog or this survey, please do not hesitate to call the Auckland SPCA on (09) 256 7300 and ask to speak to the Canine Team Leader.

* 37. Would you like someone from the SPCA to call you about any problems you have identified in this questionnaire?

- yes
- no thanks
- Not applicable

If so, what number would you like to be contacted on?
